# Implementing Caring Technologies and Social Mobilisation for Older Adults: A Mixed-Methods Evaluation Across Seven European Case Studies

**DOI:** 10.3390/ijerph23060783

**Published:** 2026-06-11

**Authors:** Toni Wright, Michelle England, Thomas Thompson, Sabina Hulbert, Theofanis Fotis, Eleni Hatzidimitriadou

**Affiliations:** 1 School of Nursing, Midwifery, Allied and Public Health, Canterbury Christ Church University, Canterbury CT1 1QU, UKtet1@hotmail.co.uk (T.T.);; 2 Centre for Health Services Studies, University of Kent, Canterbury CT2 7NF, UK; 3 School of Education, Sports & Health Sciences, University of Brighton, Brighton BN1 9PH, UK; t.fotis@brighton.ac.uk

**Keywords:** older adults, asset-based care, empowerment, self-efficacy, wellbeing, digital literacy, person-centred care

## Abstract

**Highlights:**

**Public health relevance—How does this work relate to a public health issue?**

- The research addresses the pressing issue across Europe of ageing populations and growing challenges for health and social care systems.
- It responds to current policy around the shift away from hospital to community-based care and from analogue to digital services.

**Public health significance—Why is this work of significance to public health?**

- The research adds to the growing body of knowledge concerned with how digital tools and assistive technologies can support and improve older people’s independence and self- management.
- The findings show the benefits of improved self-efficacy, mental health, digital literacy and internet-seeking behaviour from community asset-based caring technology interventions.

**Public health implications—What are the key implications or messages for practitioners, policy makers and/or researchers in public health?**

- Older people involved in initiatives aimed at building stronger community networks, reducing loneliness and fostering engagement see improved autonomy, self-management, independent living and social mobilisation.
- Research on the cost benefits of community asset-based approaches is needed if responses to the health and care of ageing populations are to provide good return on investment and be sustainable.

**Abstract:**

Population ageing presents growing challenges for health and social care systems, particularly in supporting older adults to remain independent and involved in decisions concerning their own health and wellbeing. The EMPOWERing individuals and communities to manage their own CARE (EMPOWERCARE) project evaluated asset-based initiatives designed to support older adults in managing their health and wellbeing across seven pilot sites in Belgium, France, the Netherlands and the United Kingdom. Initiatives were categorised as caring technologies, which focused on digital tools and assistive technologies to improve autonomy, promote self-management, and support independent living, and social mobilisation initiatives aimed at building stronger community networks, reducing loneliness, and fostering engagement. A multi-site, embedded case study design combined quantitative and qualitative methods. Survey data were collected at baseline (T0; n = 187) and endpoint (T2; n = 105) between July 2021 and January 2023. Outcomes included self-efficacy, mental wellbeing, loneliness and digital literacy. Descriptive statistics and repeated-measures *t*-tests were conducted, while Photovoice and focus group data were analysed using summative content analysis. Findings indicated improvements in self-efficacy and mental health among some participants, alongside positive trends in digital literacy and internet-based health-seeking behaviour. Qualitative findings further highlighted increased confidence, social connectedness and empowerment among participants.

## 1. Background

The World Health Organisation (WHO) has been documenting the increasing longevity and growing numbers of older adults for some time [1]. Regarding the health needs of older adults, there have been calls for further high-quality research to inform health policy and improve integrated care across sectors. On a community level, mobilisation aimed at tackling ageism and promoting autonomy, independence and enablement for older adults is considered key to ensuring older adults’ health needs are not overlooked and underserved [2]. This global challenge requires greater collaboration across organisations, countries, regions and communities.

There have been calls for an increase in high-quality research into older adults’ health needs which can be utilised to inform health policy, improve integrated care across sectors and inform care practice for current and future generations of older adults [3]. In this context, community-level mobilisation aimed at increasing autonomy, independence and enablement is considered key to tackling the issues of a growing and ageing population and ensuring older adults’ health needs are not overlooked and underserved [1,4]. Even where care initiatives which tackle autonomy, independence and enablement are introduced, there has been a significant lack of strong evidence that they are effective [5]. However, research in this area is developing, and this project adds to the growing evidence of the benefits that asset-based initiatives can provide for older adults’ care [6].

The EMPOWERing individuals and communities to manage their own CARE (EMPOWERCARE) social innovation aimed to address two challenges: first, the increasing longevity and growing numbers of older adults with health needs, and second, the issue of these populations not being sufficiently involved in decisions concerning their own health and wellbeing [7].

The innovation was underpinned by the EMPOWERCARE Strategy [8]. This strategy was informed by the combined asset-based work of several existing initiatives aimed at ensuring older adults are at the forefront of improved technology and better care within their communities, and empowering people to take more responsibility for their care using technology and local services to keep them motivated and in control of decisions that affect their health and wellbeing.

The idea of asset-based approaches for improving health and wellbeing has been around for over five decades; however, there is a significant lack of strong evidence that they are effective, particularly in reducing health inequities [9,10]. In asset-based approaches, such as the project initiatives, assets include social capital, self-esteem and strong communities.

Asset-based approaches are underpinned by an anti-deficit rationale. They seek to ask what benefits our health as opposed to what damages it, and work with what people can do rather than focusing on what they cannot do. They also look to build on community assets, whether those are individual, family- or community-related, to enhance people’s health and wellbeing and improve life chances [11]. There is a strong element of empowerment also underpinning such approaches. This means their purpose is to encourage and develop better self-care so that individuals and communities have the knowledge, skills and confidence to take more control and management of their own care. Sustainability is also a key attribute, with approaches such as these seeking to build on existing capacities at both the individual and community levels to sustain gains in health and wellbeing [10].

Evidence of the effectiveness of asset-based approaches is sparse and has only recently begun to emerge. This sparsity means that information about how to implement large-scale asset-based initiatives and evidence for policy change is largely absent. This paper contributes to addressing this scarcity gap. It also aligns with current policy drivers decentring hospital care in favour of community-oriented health services and the harnessing of caring technologies that empower individuals to have more control over their own health [11]. It achieves this through a unique multi-site, cross-national evaluation of an older adult health initiative set against the backdrop of the COVID-19 pandemic.

Suggestions for ways of effectively evaluating asset-based initiatives that can fill the evidence gap include asset mapping exercises, the use of national indicators as baseline measures and patient-reported outcome measures (PROMs). Standard outcome methodologies and methods are considered less useful than those from the qualitative paradigm, such as action research and appreciative inquiry [11]. Qualitative methods for evaluations geared towards discovering lived experiences are favoured, e.g., collecting narratives and stories about self-efficacy, resilience, learning and managing [11,12], and the use of visual and arts-based approaches to data collection is recommended as commensurate with exploring experiences [10].

### 1.1. Project Context

The EMPOWERCARE partnership was a European Union-funded innovation involving thirteen partners across four countries: Belgium, France, the Netherlands and the United Kingdom (UK). Partner organisations involved in the evaluation of care initiatives included community care providers, healthcare services, local authorities, academic institutions and social service agencies, each bringing expertise in supporting older adults and addressing gaps in care delivery. Collaborative cross-border visits and knowledge-sharing among partners helped shape the development and adaptation of initiatives. Project initiatives were introduced by project partners and were subsequently piloted and evaluated. By building on existing good practices and care models, the project sought to enhance these approaches, adapt them to local contexts and share results to inform future innovation and policy, emphasising person-centred approaches and caring technologies to empower older adults and support independent living [2].

The project introduced a variety of initiatives, which can be separated into two categories: caring technologies and social mobilisation. Caring technologies initiatives focused on introducing and training end-users in digital tools and assistive technologies to improve autonomy, promote self-management and support independent living. These included digital skills training, remote coaching using wearable devices, and providing access to assistive technologies such as tablets and other digital tools [13]. Social mobilisation initiatives centred on building stronger community networks, reducing loneliness and fostering engagement among older adults. Examples included developing community hubs, neighbourhood-based care networks and action plans that mobilised local resources to enhance wellbeing. Social mobilisation initiatives were conducted at one site in France (France 1) and across multiple sites in Belgium (Belgium 2 and Belgium 3). Caring technologies initiatives were implemented at single sites in Belgium (Belgium 1 and Belgium 4) and the UK (UK 1), and across multiple sites in the Netherlands (The Netherlands 1).

The COVID-19 pandemic necessitated flexibility in how the initiatives were delivered, with most being implemented remotely in line with local public health guidance. The evaluation of these initiatives, using methods such as surveys and focus groups, highlighted the interplay between technology, social engagement and care practices in addressing gaps in the support and care of older adults.

The evaluation of the project initiatives explored the ways these initiatives were effective for end-users. A multi-site embedded case study design [14] combined qualitative and quantitative data sources. Data were triangulated between surveys, focus groups and the Photovoice method.

### 1.2. Evaluation Aim and Questions

The aim of the evaluation was to evaluate the value of asset-based older adults’ health and wellbeing initiatives across seven pilot sites in four participating countries through the EMPOWERCARE project. The evaluation questions were as follows:

1. What were the lived experiences of end-users involved in the initiatives?
2. What was the impact of the initiatives on end-users in relation to mental wellbeing, loneliness, self-efficacy and digital literacy?

## 2. Materials and Methods

### 2.1. Research Design

Given the range of initiatives introduced during the project, a descriptive case study design [14] was adopted, enabling the use of multiple data collection methods; namely, a survey comprising a range of quantitative measures of pre/post outcomes, combined with qualitative data generated from a visual method: Photovoice [15] and focus group discussions (FGD). Seven pilot sites acted as independent case studies with similarities and differences synthesised at the final stage to explain the initiatives’ combined outcomes.

It was important to consider the initiative type during the analysis to reflect the area of focus each case study site had for their own interpretation of their initiative, as sites showed improvement in different areas depending on their area of focus. To determine the initiative types, two data sets were gathered as part of a case study site partner consultation: first, notes taken by the research team at online visits with case study sites, and second, case study site partners completed a survey that asked for contextual information about their initiatives and organisations.

A documentary analysis [16] of the data revealed that initiatives fell into one of two categories: caring technologies or social mobilisation. Table 1 shows examples of the 2 types of initiatives.

Evaluation data collection involved 3 time points: baseline (T0), mid-point (T1) and end-point (T2) between July 2021 and January 2023. Survey data were collected at baseline and endpoint (post-initiative) to measure the impact of the initiatives on various measures. Mid-point data collection focused on descriptive and conceptual data to inform how and why initiatives worked, using FGDs and the Photovoice method of inquiry.

### 2.2. Participants

Recruitment was conducted locally by individual partner sites and all those who took part in the initiatives were invited to take part in evaluation study. Participant demographics are reported in Table 2. Inclusion criteria for the initiatives included end-users aged 65 and older, and those aged 50 and above with at least one chronic condition (Individuals considered vulnerable, such as those living with severe mental illnesses, dementia or without capacity to consent, were not included.).

The evaluation did not employ a predefined target sample size because the EMPOWERCARE interventions were designed using an open access, “open door” approach. As an implementation project, participation was determined by local service uptake rather than researcher-controlled recruitment targets, reflecting the real-world delivery of community-based, asset-oriented initiatives. This approach prioritised inclusivity, accessibility and responsiveness to local need, allowing all eligible end-users to participate. As such, the resulting sample represents a naturalistic cohort of individuals engaging with interventions in routine practice, rather than a fixed or controlled sample.

For the survey tool, the total number of end-users recruited at T0 was 187; however, the number of end-user respondents who completed the survey at T2 was 105 (43.8% attrition). Overall, 18 end-user participants took part in the Photovoice qualitative data collection: 16 women and 2 men. Focus group discussions involved 32 end-users at the mid-point and 33 end-users at end-point spread across all sites.

### 2.3. Ethics

Ethics approval for the non-UK sites was approved by the Canterbury Christ Church University Faculty of Medicine, Health and Social Care Ethics Panel in May 2021 (reference ETH2021-0188). The Health Research Authority gave approval for the UK site in January 2022 (reference 21/WM/0200).

### 2.4. Materials

Due to COVID-19 travel and in-person restrictions, all evaluation data were collected locally by members of staff working at the case study sites. The staff received guidelines and online training in data collection techniques from the evaluation team. Quantitative data were collected using a survey tool designed by the evaluation team, containing a range of pre-validated psychometric scales, and qualitative data were collected through Photovoice [15] and FGDs. Photovoice is a community-based, participatory visual data collection method that utilises photo images taken and selected by participants to reflect upon their experiences [15].

#### Quantitative Measures

An evaluation survey tool was designed by the project evaluation team to capture the pre/post outcomes for the innovations. For this purpose, data were collected at two time points: baseline (T0), or pre-initiative and end-point (T2), or end of the initiative. These time points were the main independent (or predictor) variables and were the first level basis for comparative analysis measuring levels of association within each of the two time points and the size/direction of the association. Additional exposure variables included the country (UK, France, the Netherlands or Belgium), type of service (local authority or small organisation), type of initiative (group or individual) and model of delivery (in-person or digital). The tools were completed flexibly both online and with hard copies. Cross culturally validated psychometric scales were chosen to evaluate outcomes to ensure information was collected consistently across countries.

### 2.5. Impact of Health Status on Everyday Life

The impact of health status on everyday life was measured via the SF-12 Health Survey [17]. This is a health-related quality-of-life questionnaire which contains twelve questions organised into eight health domains relating to physical and mental health. The SF-12 generates two sub-scale scores: a mental component summary (MCS) and a physical component summary (PCS). For MSC analysis, we investigated change over time for participants with scores indicative of depression at baseline. Similarly, for the PCS analysis, we investigated change over time for participants with below-average physical health scores.

### 2.6. Mental Wellbeing

Wellbeing was measured through the shortened 7-item version of the Warwick Edinburgh Mental Wellbeing Scale (SWEMWBS) [18]. The WEMWBS was developed to enable the monitoring of mental wellbeing in the general population and the evaluation of projects, programmes and policies which aim to improve mental wellbeing. For the purpose of the evaluation analysis, we investigated change over time for participants with scores indicative of possible depression at baseline.

### 2.7. Self-Efficacy

Self-efficacy was measured through the 13-item Patient Activation Measure (PAM-13) [19], which assesses patients’ active behaviour in their self-management of chronic illnesses. For the purpose of the evaluation analysis, we investigated change over time for participants with lower levels (combined Level 1 and Level 2) at baseline.

### 2.8. Loneliness

Participants’ emotional and social loneliness was measured through the shortened version of the De Jong Gierveld Loneliness Scale [20]. The 6-item scale has been found to measure emotional and social loneliness well in multiple European countries [21]. For the purpose of the evaluation analysis, we investigated change over time for participants with scores indicative of loneliness at baseline.

### 2.9. Internet Health-Seeking Behaviour

Internet health-seeking behaviour was measured via the self-reported e-HEALS scale [22]. This 8-item scale of health literacy measures respondents’ perceived knowledge, comfort and skills at finding, evaluating and applying electronic health information in response to health problems. For the purpose of the evaluation analysis, we investigated change over time for participants with scores indicative of poor eHealth literacy at baseline.

### 2.10. Technophilia

Older adults’ attitudes and enthusiasm towards technology were measured using the 8-item Technophilia or TechPH scale [23]. Technophilia refers to a person’s enthusiasm for and positive feelings toward their technology use, alongside the absence of the fears and doubts some older adults could have about their ability to use new technology.

#### 2.10.1. Qualitative Methods

For the Photovoice element, end-users were asked to take up to 3 photographs that captured how their engagement in the project initiatives had influenced them. These pictures were to be of inanimate objects that symbolised their experiences. To ensure anonymity, end-users were advised not to take photographs of people or any items that would allow anyone to be identified. The participants were then asked to write a short written explanatory commentary to accompany each of their photographs. The photographs and commentaries produced through the Photovoice method were then used to create two online exhibitions (though in-person exhibitions did take place where COVID-19 guidance allowed). Each organisation curated exhibitions, one at the mid-point and one at the end-point which displayed the pictures and commentaries to participants. The reason for holding two consultations at both data collection points was to capture impact as the implementation of the initiatives developed. The exhibitions acted as the trigger for the FGDs.

#### 2.10.2. Data Analysis

Descriptive statistics and repeated-measures *t*-tests were conducted in IBM SPSS Statistics version 29 to examine changes in measures pre- and post-initiative.

Assumptions underlying the use of paired *t*-tests were examined prior to analysis. Although the Shapiro–Wilk test was statistically significant, it is known to be sensitive in small sample sizes and should not be used as the only evidence of deviation. Graphic inspections and indexes of kurtosis and skewness indicated no substantial deviations. To assess the robustness of the results, both paired *T*-tests and Wilcoxon signed-rank tests were conducted, and the results were largely consistent. Therefore, *T*-tests were chosen as the primary analysis with the Wilcoxon results considered as supplementary confirmation.

As partner initiatives varied in their focus, subgroup analyses were conducted by initiative type (caring technologies and social mobilisation) to examine whether outcomes differed according to the nature of the initiatives. To reduce ceiling effects, we analysed lower baseline groups for each measure to provide a more sensitive and valid estimate of initiative impact.

Photovoice data were analysed using summative content analysis, which identified the occurrence of certain words, themes and concepts. The contents were then interpreted for their underlying meaning [24].

FGD data were analysed using summative content analysis [24].

Integrated results are presented by combining survey data with findings from Photovoice and FGD to provide a fuller account of each outcome.

## 3. Results

### 3.1. Demographic Characteristics of Evaluation Participants

Demographic data included gender, age, ethnicity (demographic information was collected in accordance with local ethical guidelines), living status, educational level, and work status.

Overall, 187 end-users participated in the survey evaluation. Participants were predominantly aged between 70 and 79 (32%) and 80–89 (26%), with smaller proportions in the 60–69 (25%), 50–59 (7%) and 90–100 (11%) age bands. The majority of participants in the 90–100 age band were located in Belgium (15/21) France (4/21) and UK (2/21). A very small number (1%) preferred not to disclose their age. The majority of participants were women (67%), with men comprising 32%, while 1% identified as transgender and another 1% preferred not to specify their gender. In terms of ethnicity, 56% identified as white European, 2% as other, and a notable 42% chose not to disclose their ethnicity. Regarding living arrangements, most participants lived alone (57%), followed by those living with a partner (33%), while smaller groups lived with children (4%) or in other arrangements (6%).

### 3.2. Efficacy of Initiatives

This evaluation presents the observed changes across multiple case study sites during the initiative, by integrating both quantitative and two types of qualitative data to provide a comprehensive understanding of end-user outcomes. Quantitative results are summarised in Table 3. By integrating results from the focus group discussions and photovoice methodologies, trends in the survey data comparing pre-initiative and post-initiative measures can be understood more holistically.

#### 3.2.1. Self-Efficacy

For the analysis of self-efficacy as measured by PAM-13, we employed the recommended approach to combine Level 1 and 2 scores (indicating inactivity) and Level 3 and 4 scores (indicating activation), to assess changes from the baseline to end of the initiatives phase. Cases were adjusted for the inclusion/exclusion criteria, leaving a total sample of n = 45. Across all sites, ‘inactive’ participants at baseline (M = 48.30, SD = 4.44) showed a statistically significant increase in self-efficacy at the end of the initiatives (M = 51.10, SD = 10.72); (t = −1.909, df = 44, *p* < 0.05). This significance was not replicated in either initiative subgroup (caring technologies n = 22, *p* = 0.111; social mobilisation n = 23, *p* = 0.355).

Qualitative data offered a complementary perspective as improved self-efficacy, expressed as increased confidence and capacity, was evident from participants’ Photovoice feedback.

**Figure 1:**
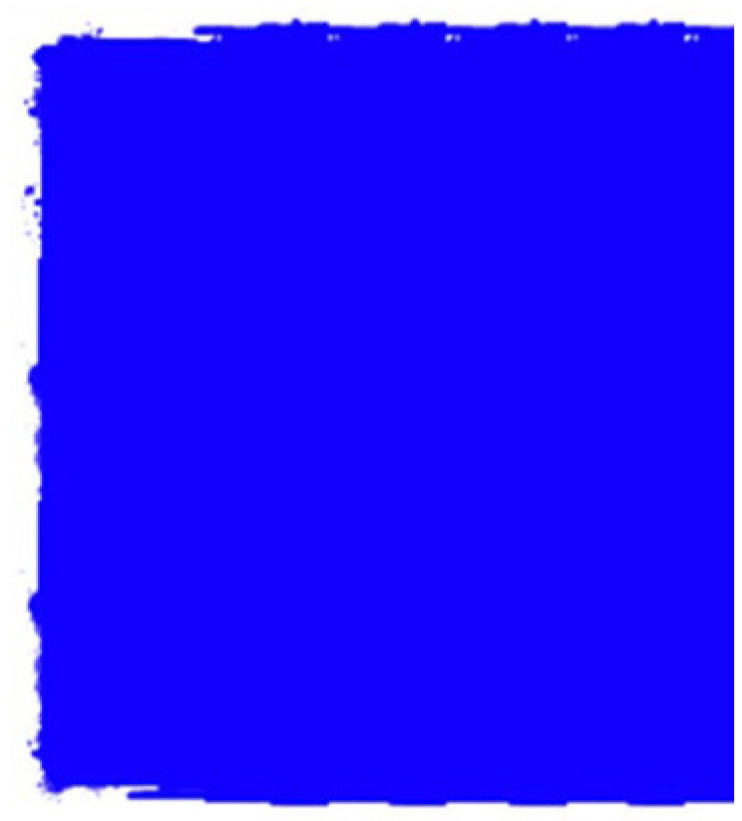
Blue square (French end-user).

An illustrative example can be seen in Figure 1, a picture from a French end-user. The end-user explains the image:

‘This blue symbolizes the sea evocative of envy to take back fishing and reconnect with my past pleasure.’

Through engagement with the programme activities, the end-user felt able to imagine and reconnect to their past and specifically with their previous hobby of fishing, thus illustrating a belief in the potential capacity to act and return to a treasured former activity.

**Figure 2:**
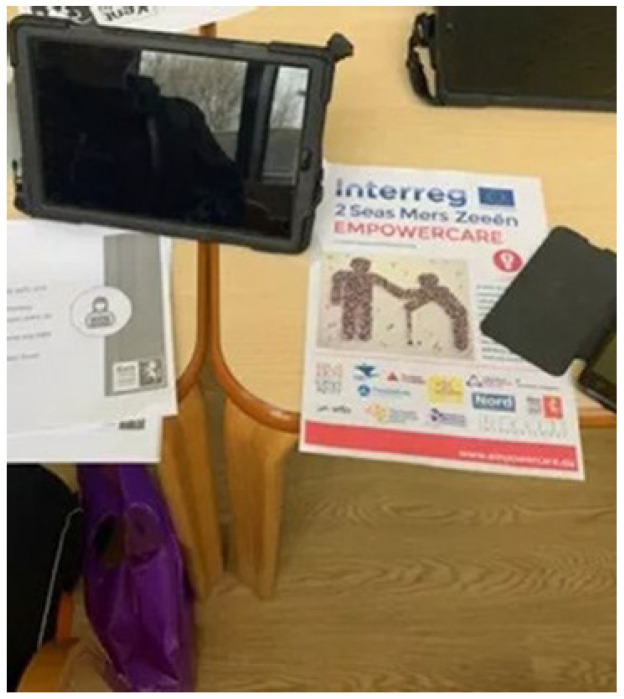
Tablet (UK end-user).

Similarly, as demonstrated by a Photovoice picture in Figure 2, a UK end-user talked about their experience of the Digital Ambassador Programme:

‘I found the sessions really helpful. They have given me more confidence to try out new apps on my phone. I enjoy using the tablet that has been loaned to me. I have also learnt about how to recognise a scam and what to do if I receive a scam email or message on Facebook.’

(UK end-user)

This quote illustrates a functional improvement in digital skills, as well as an increase in self-perceived agency. This is a concrete example of how an end-user had been enabled to feel confident in their technological abilities.

#### 3.2.2. Emotional and Social Wellbeing

Across all sites, cases were adjusted for inclusion/exclusion criteria, with a total sample of 50 for the MCS and 63 for the PCS. Participants with scores indicative of depression at baseline (M = 21.93, SD = 3.05), reported a statistically significant improvement at the end of the initiatives (M = 36.86, SD = 5.82) (t = −5.271, df = 49, two-tailed *p* < 0.001). This was driven by social mobilisation participants (n = 31). Social mobilisation participants with scores indicative of a depressive disorder at baseline (T0) (M = 35.74, SD = 6.05), showed a statistically significant improvement at the end of initiatives (T2) (M = 45.24, SD = 9.55) (t = −5.569, df = 30, *p* < 0.001). This significance was not reported in the caring technologies subgroup (n = 19, *p* = 0.125). This suggests that the social nature of these initiatives played a crucial role in improving mental health, an aspect that was less pronounced in the technology-driven initiatives.

Participants who reported a below-average physical health score for their age, showed a small non-significant increase in mean scores from baseline (T0) (M = 33.01, SD = 6.43) to the end of initiatives (M = 34.99, SD = 9.11); (t = −1.961, df = 62, *p* = 0.054). Neither subgroup showed a significant difference post-initiative. Restrictions on movement, social distancing and reduced access to healthcare or physical activity programmes according to COVID-19 health guidance could have limited participants’ ability to engage in regular exercise, particularly in older populations or those with chronic health conditions, although this cannot be confirmed from the current data.

Feedback from FGDs adds more context to the survey results, as illustrated below:

‘I find it hard to take time and I realised that I needed to take. This was a time for me to find out what we could do. Doing sport is good for you. I don’t do sport anymore because I don’t feel like it but (anonymised name’s) activities have rekindled the spark and I’ve taken up swimming again in the same time slot as X. If he smiles, I smile.’

(French end-user)

##### Mental Wellbeing

Across all sites, cases were adjusted for inclusion/exclusion criteria, leaving a total sample of (n = 83), participants who reported either average or low mental wellbeing at baseline reported no significant difference at the end of the initiatives (T0: M = 21.93, SD = 3.05; T2: M = 22.02, SD = 3.37; t = −0.261, df = 82, *p* = 0.795). Social mobilisation participants with low or average mental wellbeing scores at baseline also reported no significant differences at the end of the initiatives. Caring technologies participants showed a statistically significant decrease in mental wellbeing scores (T0: M = 22.37, SD = 2.10; T2: M = 21.56, SD = 2.51; t = 2.284, df = 34, *p* = 0.029). However, a corresponding Wilcoxon signed-rank test indicated a similar trend but did not reach statistical significance (*Z* = −1.855, *p* = 0.064).

**Figure 3:**
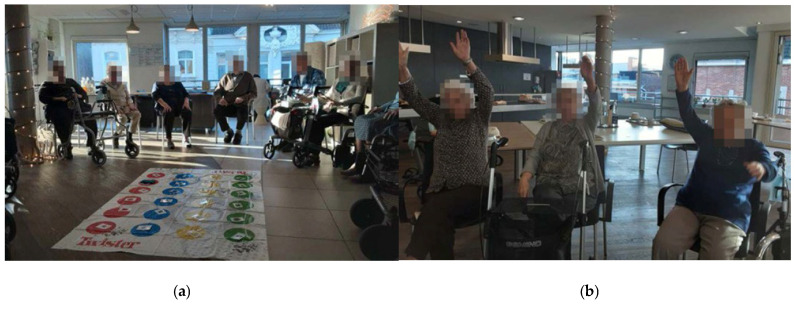
(**a**,**b**) Group movement games (Belgian end-user, social mobilisation).

Alternatively, qualitative feedback showed that for those who participated in group activities (Figure 3a,b) there was a positive affect:

‘The combination between social contact and moving and getting to know fellow residents in a different way exercise in a group gives me energy and perseverance.’

(Belgian end-user)

During an FGD another end-user said of the initiative in Belgium:

‘I feel happier and can do more independently’.

Data on social and emotional wellbeing suggest that the project initiatives had a positive impact on end-users. The following focus group discussion quotations encapsulate this:

‘I’ve set many things in motion. I built a life for myself, relationships with the outside world, people who helped me to open up, to meet people.’

(French end-user)

‘I take care together with sister. Since she has been here, the care is better and she is flourishing again, daring to do things herself that she was no longer doing at home.’

(Belgian community stakeholder)

Not all pictures evoked positive comments, however. The FGDs in Belgium provided insight into how end-users felt there were times when more could be done to support them. One end-user reflecting on the Photovoice exhibition, noted that a picture was unclear and dark, and it evoked negative thoughts affecting their mental wellbeing:

‘I like to have a lot of natural light/lux light in the living room of my floor, it has a great influence on my mood. I find that there is little light in the living room on floor 2. Often dark in my living room, I need a lot of daylight. In the winter period this gives a dark impression which affects my mood.’

(Belgian end-user)

Overall, qualitative data suggests positive outcomes for end-users’ mental wellbeing, as participants reported that the initiatives had enabled greater autonomy and freedom.

#### 3.2.3. Loneliness

When considering all project sites, cases were adjusted for inclusion/exclusion criteria, leaving a total sample of 95. There was no significant change to the level of reported loneliness, overall and by type (emotional and social). Subgroup analysis also revealed that participants from the social mobilisation (n = 60) and caring technologies (n = 35) initiatives experienced no significant change in loneliness overall and by type. This may reflect the well-documented effects on loneliness during the COVID-19 data collection period.

Findings from the qualitative data analysis provided a different insight into the participants’ experiences of loneliness. In fact, the analysis demonstrated how the initiatives had a positive impact in reducing the loneliness levels of end-users.

**Figure 4:**
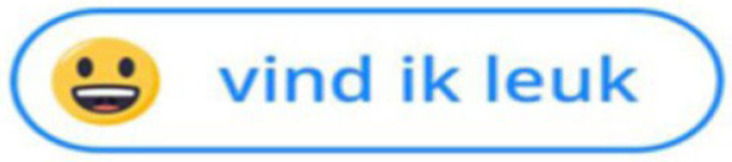
Happy emoji (Dutch end-user).

For example, a Dutch end-user chose an emoji image (Figure 4) with wording that translates as ‘I like this’, stating the positive impact of being part of the initiative:

‘I don’t really have a personal goal. Taking part in the digital activities is recreating for me. I am enjoying meeting other people, I need it. Sometimes it is very quiet at home’.

(Dutch end-user)

Likewise, a French FGD participant commented on the significance of being involved in the project initiative:

‘It helped me to get out of my loneliness. My doctor told me ‘You mustn’t stay like that’, but he didn’t tell me what to do. I even went so far as to think about suicide. Maybe I would have done that. I wouldn’t be here today. Now I know I can count on someone; it breaks my isolation. I can call my nurse, the young people from Unicity, my housekeeper. I can count on these people. I have a little list in my notebook.’

(French end-user)

This quote is very arresting and illustrates a real depth of loneliness that some participants experienced.

Both testimonies demonstrate the sense of loneliness end-users felt pre-initiative and the positive effect the initiative had in tackling it.

#### 3.2.4. End-Users’ Digital Literacy

##### Internet Health-Seeking Behaviour

When analysing survey responses from all sites, cases were adjusted for inclusion/exclusion criteria, leaving a total sample of 97. End-users reported a small, non-significant increase in internet health-seeking behaviour at the end of the initiatives (T0: M = 23.41, SD = 7.61; T2: M = 22.46, SD = 7.64; t = 1.533, df = 96, *p* = 0.129). Caring technology initiatives reported no significant change in internet health-seeking behaviour post-initiative (n = 62, *p* = 0.23). In contrast, social mobilisation initiatives reported a significant decrease in internet health-seeking behaviour T0: M = 22.67, SD = 6.88; T2: M = 20.66, SD = 7.32; t = 2.619, df = 57, *p* < 0.05).

Qualitative data from end-users supported the survey results of those participating in caring technologies initiatives. They reported feeling more digitally literate and confident in using technology for their physical and mental health needs and to keep connected with their social networks.

**Figure 5:**
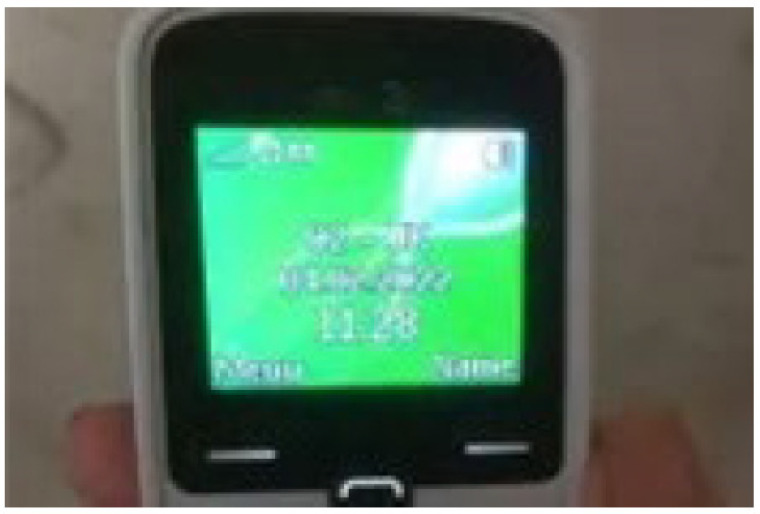
Mobile phone (UK end-user).

Figure 5, from the Photovoice data helps to determine this, with a UK end-user commenting positively on how his digital skills developed during the initiative:

‘I feel more confident using the phone and I’ve learned to do a lot more, for instance use capitals, and the torch. That was really good the night we went to the concert. Confident receiving and sending texts. I didn’t realise before that messages waited in the outbox—that I had to scroll down and press send, so I had messages I thought I’d sent, but they hadn’t gone, but now I know what to do. I couldn’t read what I’d sent but now I can.’

(UK end-user)

**Figure 6:**
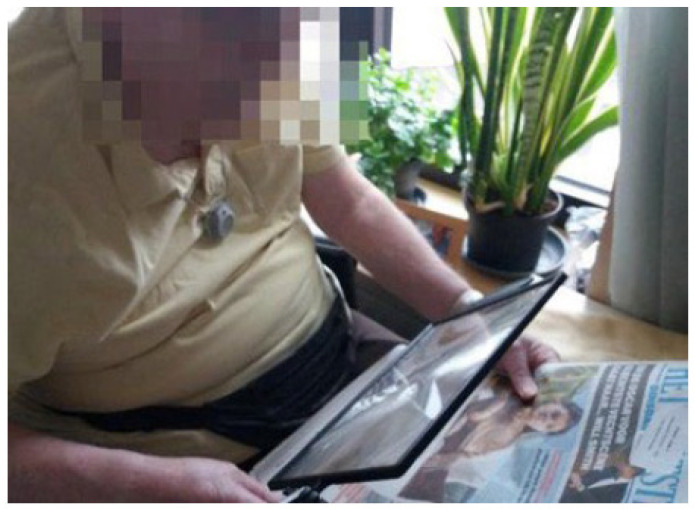
Reading loop (Belgian end-user).

Similarly, a Belgian end-user chose an image of a reading loop (Figure 6) and noted: ‘I like to be up to date with current events, independent, reading and action group.’

In the same vein, a French focus group participant stated: ‘People around me were happy to see me getting interested in digital technology and taking pictures and sharing them on the application.’

Overall, the qualitative digital literacy data showed improvements in digital literacy for end-users, demonstrating how this improvement enabled them to share experiences with others, feel independent and autonomous and connect with other people.

#### 3.2.5. Technophilia

When analysing survey responses across all sites (Cases were adjusted for inclusion/exclusion criteria, leaving a total sample of 97), there were no significant changes in reported tech-enthusiasm (*p* = 0.603), tech-anxiety (*p* = 0.245), or tech index (*p* = 0.32).

Participants in caring technologies initiatives (n = 35, *p* = 0.32) and social mobilisation (n = 62, *p* = 0.959) showed no meaningful change in tech index scores post-initiative.

Caring technologies (n = 35) participants did not show any significant change in tech-enthusiasm (*p* = 0.406) and tech-anxiety (*p* = 0.738). Social mobilisation (n = 62) participants did not show any significant change in tech-enthusiasm (*p* = 0.243) and tech-anxiety (*p* = 0.235). This finding raises important questions about the deeper psychological and emotional factors that shape technology adoption among older adults.

**Figure 7:**
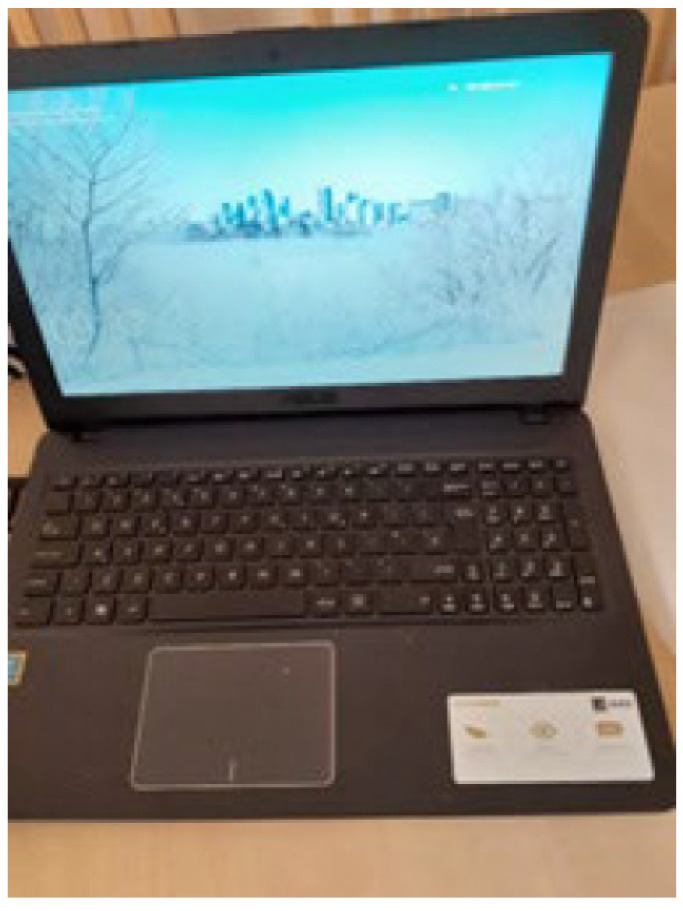
Laptop (UK end-user).

Photovoice data offered further insight into end-users’ attitudes towards technology. For example, a UK based end-user who commented on their picture of the laptop they used during the initiative (Figure 7) said:

‘During my time with you, I have worked on emails with additional pictures and Microsoft Office, creating columns for library work. I feel that the computer work is going to get harder, and I am not going to be able to carry out the tasks set. I still lack so much confidence and it worries me.’

(UK end-user)

Most end-users who participated in the Photovoice exercise expressed that they had gained confidence from learning more about how to use technology, but there were others who felt confidence was something that still needed to be built on. The caption for Figure 7 is an expression of trepidation that gives an interesting insight into feelings of apprehension for older adults around living in a digital/computer age. It is worth noting that navigating a laptop can be more complex than a non-smart mobile phone or a reading loop and so learning how to use it may be a more daunting prospect.

Overall, qualitative feedback from participants on technology was positive, with end-users noting the benefits to wellbeing and independence stemming from their increased technological confidence and knowledge. This highlights the potential of digital initiatives to enhance autonomy but also underscores the need for tailored support. Two French focus group discussion participants exemplify this:

‘I didn’t dare tell my son that I wasn’t well. My son sensed that something was wrong. It was difficult for me to ask; I didn’t want to disturb him. I finally told him and now he texts me or I hear my great- granddaughter on the phone going ‘areuh’ and it brightens up my whole week.’

(French end-user)

‘My tablet is becoming a drug. Every day I send a note to my grandson. My wish was to go to Montreal to see my grandson, which will happen in September with my son.’

(French end-user)

## 4. Discussion

The EMPOWERCARE project evaluation aimed to illuminate end-users’ lived experiences by synthesising quantitative and qualitative findings on the initiatives’ impact on self-efficacy, mental wellbeing, loneliness and internet health-seeking behaviour, producing a mixed picture of outcomes across initiative types.

Findings of the evaluation study confirmed that the project initiatives and approach had key positive outcomes for all participants. First, participants at baseline showed significant positive changes in self-efficacy, as triangulated by both quantitative and qualitative findings, although this finding should be interpreted with caution as significance was not replicated in either subgroup. Qualitative participant feedback suggested that the initiatives supported end-users to have increased levels of self-confidence and self-efficacy around taking action in relation to their own health.

Mental health improved significantly overall, particularly in the social mobilisation initiatives, although not in the caring technologies initiatives. Physical health showed a small non-significant trend, but overall, no significant change was observed. The nature of research on older adults and adults with chronic conditions, where ageing continues throughout the research process, coupled with the reduction in physical activity available during COVID-19 restrictions, could explain this lack of significance. Alternatively, qualitative data reported a reignition of a spark in physical activity.

Mental wellbeing reported no significant change over the total sample or within the social mobilisation initiatives. There was however a significant decrease in wellbeing within the caring technologies initiatives. The corresponding Wilcoxon signed-rank test showed a similar trend but did not reach significance, possibly due to the sample size. Quantitative data suggest that project initiatives had a positive impact on end-users, enabling greater autonomy and freedom, although the qualitative data also report that end-users felt there were times when more could be done to support them.

Survey results did not show a statistically significant decrease in self-reported loneliness, both in the overall sample and by initiative type. It is worth noting that the initiatives and data collection took place during COVID-19 lockdown measures. The social distancing, especially with older and vulnerable populations, could have skewed the quantitative data results. While COVID-19 lockdown measures were found to significantly increase loneliness [25], and restrictions may have counteracted potential benefits, the absence of a significant decrease suggests that the initiatives could have provided protective opportunities for individuals. On the other hand, qualitative analysis suggested a positive impact the initiatives had on end-users’ loneliness, as participants reported an enhanced sense of autonomy and social connectedness.

Qualitative feedback from end-users suggests that those participants felt more digitally literate and confident in using technology for their physical and mental health needs, as well as to keep connected with their social networks. Quantitative results did not reveal significant positive changes, and social mobilisation initiatives were even associated with a significant decrease in digital health literacy post-initiative. This raises the question of whether the caring technology initiatives provided a protective factor against a natural decrease in digital literacy over time.

There were no significant changes reported in technology-enthusiasm, technology-anxiety or the combined score across either initiative type. Qualitative results suggest that some end-users reported benefits to wellbeing and independence stemming from their increased technological confidence and knowledge. However, some participants still felt a lack of confidence in using technology.

Funding for the project ended in 2023, but the initiatives have continued. Notably, in the UK, digital literacy programmes for older adults have been adopted by the local council involved in the project and are ongoing, and in Belgium more digital devices have been purchased by the project partner organisations to support and sustain both service delivery and service growth.

The implementation of these initiatives during the COVID-19 pandemic, and the need to socially distance and stay at home, fostered a period of awakening about the benefits of harnessing caring technologies. It also highlighted the need for vulnerable populations, including older people, to have greater access to caring technologies and to be empowered toward better digital health literacy [26]. The escalated learning that came about because of the pandemic restrictions led to an understanding of the initiatives’ applicability to other populations. This includes the potential to meet the needs of those living with long-term conditions (LTCs) such as heart disease, chronic obstructive pulmonary disease, type-2 diabetes, neurological diseases and asthma. People living with LTCs represents a growing population globally and digital health is recognized as a potential solution to meeting their needs and supporting their health autonomy [27]. Under-served and over-looked populations, such as racialised minority, low-income, sexual and gender minority populations could also benefit from greater access to empowering caring technologies [3].

### Limitations

The evaluation highlighted the successful delivery of the project initiatives across all case study sites, although several challenges were encountered. The evaluation data were collected when all participating countries were working within the restrictions of government-instructed social measures to prevent the spread of COVID-19, which, particularly regarding the loneliness outcome, could have altered the results. In the United Kingdom and Belgium, residential care facilities could be cohorted into smaller units, thereby reducing disruption; meanwhile community-based initiatives in Belgium and the Netherlands could only operate if limited to small, consistent groups. In France and the Netherlands, digital initiatives were less affected as interactions could shift online, although in-person groups or one-to-one sessions remained constrained by social distancing measures. These restrictions also meant that the delivery sites collected data themselves under the evaluation team’s instruction, which led to some difficulties in assuring the data were collected according to the data collection plan. Working remotely and managing international delivery sites without experienced researchers meant that numerous clarifications were required, and language barriers prevented efficient data collection at points.

Due to local ethical guidelines regarding demographic data collection in selected countries, we were limited in the comparisons that could be made depending on end user demographics.

Furthermore, the project initiatives were not standardised and were interpreted and implemented differently at each case-study site. Although they were designed with the EMPOWERCARE core principles in mind, the sites covered different countries and had multiple target end-users and goals.

## 5. Conclusions

Despite the complexities of having data from different case-study sites, countries and target end-users, multiple perspectives and data sources enriched the understanding of the EMPOWERCARE initiatives. Quantitative and qualitative data results complemented each other well, demonstrating key areas where the project initiatives had a positive impact.

Insights gained during the implementation of the EMPOWERCARE for end-users showed the key areas of impact were increased self-efficacy, including a sense of enablement around their capacity to take action in relation to their own health, wellbeing and confidence in their technological abilities. Participants reported strong testimonies about the positive effect the initiatives had in tackling isolation and solitude, although these findings were not reflected in the quantitative measures. Qualitative results suggested improved digital literacy and confidence in using technology for their physical and mental health needs and in keeping connected with their social networks, although the quantitative data showed there were significant decreases in digital literacy for the social mobilisation initiatives.

The evaluation results provide some positive results regarding the success of the project initiatives across the four countries. Combining the asset-based work of existing partner initiatives from across Belgium, France, the Netherlands and the UK, the project provides preliminary evidence that asset-based approaches to older population health needs can enable older adults to achieve better health and wellbeing. Furthermore, these approaches empower them to take more responsibility for their care through the use of caring technologies and local assets, keeping them motivated to have control over decisions that affect their health and wellbeing.

With further research needed into the impact of asset-based initiatives, future work should focus on causal explanations of how and why the project outcomes occurred. To this end realist approaches have the potential to be particularly useful in unearthing underlying causes. Finally, additional research on the cost-benefits of asset-based approaches is imperative if responses to ageing populations are to provide a good return on investment and be sustainable.

## Figures and Tables

**Table 1:** Examples of caring technologies and social mobilisation initiatives.

Site	Social Mobilisation	Caring Technologies
One site	Community hub and neighbourhood care network to reduce loneliness among older adults (France)	Digital skills training and access to assistive technologies to support independent living (Belgium) Remote coaching with wearable devices to promote self-management (Belgium) Provision of tablets and digital tools to support independent living (UK)
Multiple sites	Neighbourhood-based care networks and action plans mobilising local resources for wellbeing (Belgium) Community engagement and action planning to foster social inclusion among older adults (Belgium)	Remote coaching using wearable devices and digital tools to promote self-management (The Netherlands)

**Table 2:** Participant demographic information.

		Country								
		Belgium	France			The Netherlands		United Kingdom		Total
		*N* = 104	*N* = 33			*N* = 28		*N* = 30		195
Age (Bands)	50–59	6	6%	-	-	7	25%	-	-	13
	60–69	18	17%	10	30%	6	21%	14	47%	48
	70–79	30	29%	11	33%	10	36%	11	37%	62
	80–89	35	34%	8	24%	5	18%	2	7%	50
	90–100	15	14%	4	12%	-	-	2	7%	21
	Prefer not to say	-	-	-	-	-	-	1	3%	1
Gender	Woman	72	69%	20	61%	17	61%	21	70%	130
	Man	32	31%	12	36%	11	39%	8	27%	63
	Trans	-	-	1	3%	-	-	-	-	1
	Prefer not to say	-	-	-	-	-	-	1	3%	1
Ethnicity *	White European	55	53%	-	-	27	96%	28	93%	110
	Other	1	1%	-	-	1	4%	1	3%	3
	Didn’t want to say	48	46%	33	100%	-	-	1	3%	82
Sexual Orientation *	Heterosexual/ straight	47	45%	-	-	24	86%	29	97%	100
	Prefer not to say	8	8%	-	-	3	11%	1	3%	12
	Did not answer	49	47%	33	100%	1	4%	-	-	83
Living Arrangements	Partner	36	35%	8	24%	11	39%	10	33%	65
	Children	5	5%	1	3%	-	-	1	3%	7
	Alone	58	56%	20	61%	15	54%	19	63%	112
	Other	5	5%	4	12%	2	7%	-	-	11

* Denotes the demographic characteristic was not included in the survey for French participants following local research ethics requirements.

**Table 3:** Comparisons of observed changes in evaluation quantitative results at pre (T0) and post (T1) initiative phase.

Measure	Group	n	Mean T0 (SD)	Mean T2 (SD)	t	df	*p* Value
Self-efficacy (PAM-13)	Total	45	48.30 (4.44)	51.10 (10.72)	−1.909	44	<0.031 *
Self-efficacy (PAM-13)	Caring technologies	22	49.54 (3.33)	53.60 (11.53)	−1.664	21	0.111
Self-efficacy (PAM-13)	Social mobilisation	23	47.12 (5.08)	48.70 (9.52)	−0.944	22	0.355
Mental health (MCS-12)	Total	50	21.93 (3.05)	36.86 (5.82)	−5.271	49	<0.001 *
Mental health (MCS-12)	Caring technologies	19	38.67 (5.07)	41.42 (7.55)	−1.609	18	0.125
Mental health (MCS-12)	Social mobilisation	31	35.75 (6.05)	45.25 (9.55)	−5.569	30	<0.001 *
PCS-12 physical health	Total	97	39.58 (6.43)	34.99 (9.11)	−1.961	62	0.054
PCS-12 physical health	Caring technologies	27	34.52 (6.71)	36.50 (10.79)	−1.089	26	0.286
PCS-12 physical health	Social mobilisation	36	31.87 (6.07)	33.85 (7.58)	−1.724	35	0.093
Mental wellbeing (SWEMWBS)	Total	48	21.93 (3.05)	22.02 (3.37)	−0.261	82	0.795
Mental wellbeing (SWEMWBS)	Caring technologies	35	22.37 (2.10)	21.56 (2.51)	2.284	34	0.029 *
Mental wellbeing (SWEMWBS)	Social mobilisation	48	21.62 (3.58)	22.35 (3.87)	−1.559	47	0.126
Loneliness (De Jong total)	Total	95	4.33 (1.51)	4.48 (1.53)	−1.115	94	0.268
Loneliness (De Jong total)	Caring technologies	35	4.26 (1.42)	4.54 (1.31)	−1.221	34	0.23
Loneliness (De Jong total)	Social mobilisation	60	4.38 (1.57)	4.45 (1.65)	−0.419	59	0.677
Loneliness (De Jong Emotional)	Total	95	2.04 (0.87)	2.08 (0.91)	−0.476	94	0.635
Loneliness (De Jong Emotional)	Caring technologies	35	1.86 (0.88)	2.03 (0.82)	−1.099	34	0.280
Loneliness (De Jong Emotional)	Social mobilisation	60	2.15 (0.86)	2.12 (0.96)	0.314	59	0.755
Loneliness (De Jong Social)	Total	95	2.29 (0.96)	2.40 (0.89)	−1.043	94	0.300
Loneliness (De Jong Social)	Caring technologies	35	2.40 (0.98)	2.51 (0.82)	−0.612	34	0.545
Loneliness (De Jong Social)	Social mobilisation	60	2.23 (0.95)	2.33 (0.93)	−0.847	59	0.401
Digital literacy (eHEALS)	Total	97	23.41 (7.61)	22.46 (7.64)	1.533	96	0.129
Digital literacy (eHEALS)	Caring technologies	35	23.00 (7.62)	24.26 (6.99)	−1.223	34	0.23
Digital literacy (eHEALS)	Social mobilisation	62	22.67 (6.89)	20.65 (7.32)	2.619	57	0.011 *
Tech enthusiasm	Total	97	2.31 (0.90)	2.27 (0.78)	0.521	96	0.603
Tech enthusiasm	Caring technologies	35	2.22 (0.67)	2.32 (0.65)	−0.842	34	0.406
Tech enthusiasm	Social mobilisation	62	2.37 (1.00)	2.25 (0.86)	1.18	61	0.243
Tech anxiety	Total	97	1.77 (0.69)	1.87 (0.66)	−1.169	96	0.245
Tech anxiety	Caring technologies	35	1.88 (0.66)	1.93 (0.54)	−0.337	34	0.738
Tech anxiety	Social mobilisation	62	1.71 (0.71)	1.83 (0.72)	−1.2	61	0.235
Technophilia (overall index)	Total	97	2.04 (0.57)	2.07 (0.51)	−0.573	96	0.568
Technophilia (overall index)	Caring technologies	35	2.05 (0.44)	2.12 (0.37)	−1.009	34	0.32
Technophilia (overall index)	Social mobilisation	62	2.04 (0.64)	2.04 (0.58)	−0.052	61	0.959

* denotes significance i.e., *p* < 0.05.

## Data Availability

The raw data supporting the conclusions of this article will be made available by the authors on request.

## References

[1] National Institute on Aging, National Institutes of Health, World Health Organization (2011). Global Health and Aging.

[2] World Health Organization (2017). Global Strategy and Action Plan on Ageing and Health.

[3] Khan H.T.A., Addo K.M., Findlay H. (2024). Public health challenges and responses to the growing ageing populations. Public Health Chall..

[4] Veras D.C., Lacerda G.M., Forte F.D.S. (2022). Elderly people social groups as a tool for health empowerment: Action research. Interface.

[5] Ward S. (2023). Using theory-based evaluation to understand what works in asset-based community development. Community Dev. J..

[6] Corrigan O., Hughes S., Danielsen S., Doherty S., Kabir R. (2023). The impact of engaging with community groups: Asset-based approaches and the lived experience of socially vulnerable populations in the UK. Front. Public Health.

[7] Killgore W.D.S., Cloonan S.A., Taylor E.C., Lucas D.A., Dailey N.S. (2020). Loneliness during the first half-year of COVID-19 lockdowns. Psychiatry Res..

[8] Hatzidimitriadou E., Wright T., England M., Thompson T., Lynch M. (2023). EMPOWERCARE: EMPOWERing Individuals and Communities to Manage Their Own CARE.

[9] Wright T., Hatzidimitriadou E., Stirrup V., Thompson T., DeBraal P., Burton C., Chung P., Kuzbit P., Price A., Stein M. (2022). EMPOWERing older adults and their communities to manage their own CARE (EMPOWERCARE): Evaluation study of a social innovation initiative across four European countries. Int. J. Integr. Care.

[10] Daly M., Westwood S. (2018). Asset-based approaches, older adults and social care: An analysis and critique. Ageing Soc..

[11] Al Meslamani A.Z. (2024). Why are digital health policies crucial?. J. Med. Econ..

[12] Rippon S., Hopkins T. (2015). Head, Hands and Heart: Asset-Based Approaches in Health Care.

[13] Foot J., Hopkins T. (2010). A Glass Half-Full: How an Asset Approach Can Improve Community Health and Well-Being.

[14] Foot J. (2012). What Makes Us Healthy? The Asset Approach in Practice: Evidence, Action, Evaluation.

[15] Honninx E., van Schyndel C., Roos A., Paulding E., Wright T., Galvin K., Fotis T., Huber J., Laes E., Lambrechts N. (2026). Towards caring technologies in older adult care through the co-creation of an ethical process guide. Int. J. Environ. Res. Public Health.

[16] Yin R.K. (2013). Case Study Research: Design and Methods.

[17] Wang C., Burris M.A. (1997). Photovoice: Concept, methodology, and use for participatory needs assessment. Health Educ. Behav..

[18] Gorsky M., Mold A., Pope C., Mays N. (2020). Documentary analysis. Qualitative Research in Health Care.

[19] Ware J.E., Kosinski M., Keller S.D. (1996). A 12-item short-form health survey: Construction of scales and preliminary tests of reliability and validity. Med. Care.

[20] Stewart-Brown S., Tennant A., Tennant R., Platt S., Parkinson J., Weich S. (2009). Internal construct validity of the Warwick-Edinburgh Mental Well-being Scale (WEMWBS): A Rasch analysis using data from the Scottish Health Education Population Survey. Health Qual. Life Outcomes.

[21] Hibbard J.H., Mahoney E.R., Stockard J., Tusler M. (2005). Development and testing of a short form of the patient activation measure. Health Serv. Res..

[22] De Jong-Gierveld J., Kamphuis F. (1985). The development of a Rasch-type loneliness scale. Appl. Psychol. Meas..

[23] De Jong-Gierveld J., Van Tilburg T. (2010). The De Jong Gierveld short scales for emotional and social loneliness: Tested on data from seven countries in the UN Generations and Gender Surveys. Eur. J. Ageing.

[24] Norman C.D., Skinner H.A. (2006). eHEALS: The eHealth literacy scale. J. Med. Internet Res..

[25] Anderberg P., Eivazzadeh S., Berglund J.S. (2019). A novel instrument for measuring older adults’ attitudes toward technology (TechPH): Development and validation. J. Med. Internet Res..

[26] Christie R., Sadler E., Sait M., Light K., Cox C., Board M., Thomas S., Walker D.-M., Allen-Pick M., Bradbury K. (2024). Digital empowerment in long-term condition management: A systematic review and narrative synthesis of the experiences and perceptions of home-based digital health coaching interventions. Digit. Health.

[27] Richardson S., Lawrence K., Schoenthaler A.M., Schwartz R., Feldman J., Trinh-Shevrin C. (2022). A framework for digital health equity. npj Digit. Med..

